# The Kinase USK1 Regulates Cellulase Gene Expression and Secondary Metabolite Biosynthesis in *Trichoderma reesei*

**DOI:** 10.3389/fmicb.2020.00974

**Published:** 2020-05-20

**Authors:** Sabrina Beier, Wolfgang Hinterdobler, Alberto Alonso Monroy, Hoda Bazafkan, Monika Schmoll

**Affiliations:** Center for Health and Bioresources, AIT Austrian Institute of Technology GmbH, Tulln, Austria

**Keywords:** *Trichoderma reesei*, *Hypocrea jecorina*, sorbicillin, dihydrotrichotetronin, secondary metabolism, plant cell wall degradation, signal transduction

## Abstract

The complex environment of fungi requires a delicate balance between the efforts to acquire nutrition, to reproduce, and to fend off competitors. In *Trichoderma reesei*, an interrelationship between regulation of enzyme gene expression and secondary metabolism was shown. In this study, we investigated the physiological relevance of the unique YPK1-type kinase USK1 of *T. reesei*. *Usk1* is located in the vicinity of the SOR cluster and is involved in regulation of several genes from this secondary metabolite cluster as well as dihydrotrichotetronine and other secondary metabolites. Moreover, USK1 is required for biosynthesis of normal levels of secondary metabolites in liquid culture. USK1 positively influences cellulase gene regulation, secreted cellulase activity, and biomass formation upon growth in constant darkness on cellulose. Positive effects of USK1 on transcript abundance of the regulator of secondary metabolism, *vel1*, and the carbon catabolite repressor gene *cre1* are in agreement with these functions. In summary, we found that with USK1, *T. reesei* comprises a unique kinase that adds an additional layer of regulation to the connection of secondary metabolism and enzyme production in fungi.

## Introduction

*Trichoderma reesei* (syn. *Hypocrea jecorina*) represents one of the most important filamentous fungi for industrial applications (Bischof et al., 2016). *T. reesei* is a model organism for regulation of plant cell wall degradation due to its efficient machinery for regulation and secretion of carbohydrate active enzymes (Saloheimo and Pakula, 2012; Gupta et al., 2016; Paloheimo et al., 2016). Plant cell wall degrading enzymes are largely co-regulated in *T. reesei* (Foreman et al., 2003) and their expression is regulated by a network of transcription factors (Benocci et al., 2017), of which XYR1, ACE3, and the carbon catabolite repressor CRE1 are most important. Besides transcription factors, also several signal transduction pathways impact cellulase regulation and especially light as a signal has a profound influence on enzyme expression (Schmoll, 2018).

*Trichoderma reesei* has a long tradition of application in industry and received generally regarded as safe (GRAS) status (Nevalainen et al., 1994). Recently, the safety of *T. reesei* as an industrial producer of food and feed enzymes has been re-assessed with a very low level of risk for contamination with harmful toxins (Frisvad et al., 2018). In recent years, secondary metabolism of *T. reesei* became an interesting field of investigation and also some interconnections with enzyme production became obvious, highlighting the importance of secondary metabolite screening done routinely for industrial production strains.

Sorbicillinoids represent a group of secondary metabolites produced by filamentous fungi, for which anti-inflamatory and antimicrobial functions are reported (Cram, 1948; Maskey et al., 2005) and potential anticancer and anti-HIV applications are discussed (Bringmann et al., 2003; Yao et al., 2015). The biosynthetic genes responsible for production were first elucidated in *Penicillium chrysogenum* (Salo et al., 2016; Guzman-Chavez et al., 2017). In *T. reesei*, the production of sorbicillin compounds was investigated at the molecular level. The gene cluster responsible for production of the respective yellow pigments (SOR cluster) is regulated by the two transcription factors YPR1 and YPR2 as well as the carbon catabolite repressor CRE1 and the secondary metabolism regulator LAE1 (Derntl et al., 2016, 2017a; Monroy et al., 2017). The cluster arose in early Hypocreales and lateral gene transfer led to the structure in the *T. reesei* genome (Druzhinina et al., 2016). The produced sorbicillinoids exert a growth limiting effect on other microbes (Derntl et al., 2017a). Regulation of the SOR cluster is altered in response to the carbon source (Derntl et al., 2016; Dattenböck et al., 2018; Hitzenhammer et al., 2019) and on cellulose a negative feedback cycle is obvious upon growth in light and a positive cycle in darkness (Monroy et al., 2017). Previously, also high level constitutive production of sorbicillinoids was achieved by random mutagenesis of *T. reesei* RutC30 (Li et al., 2018). However, the regulatory basis for this effect remains to be investigated.

Phosphate residues, attached to and removed from regulatory proteins in a precisely orchestrated manner, represent the currency of signaling processes within the cell. Thereby, biological activity, subcellular localization, half-life, and posttranscriptional modifications as well as interactions with other proteins are regulated (Cohen, 2000; Kosti et al., 2010; Turra et al., 2014). In fungi, diverse physiological functions are known for protein kinases and protein phosphatases including modulation of development, expression of plant cell wall degrading enzymes, secondary metabolism, as well as circadian rhythmicity and light response (Diernfellner and Schafmeier, 2011; Park et al., 2011; Ghosh et al., 2014; de Assis et al., 2015; Winkelströter et al., 2015). Recently, an involvement of different protein phosphatases in many of these mechanisms was shown for *T. reesei* as well (Rodriguez-Iglesias and Schmoll, 2019). The genome of *T. reesei* comprises 103 predicted protein kinase genes (Schmoll et al., 2016). Phosphoproteomic analysis of the reaction of *T. reesei* to inducing conditions provided insights into a complex signaling network, with phosphorylation changes in proteins associated with light-mediated cellulase regulation, carbon sensing, and osmoregulation as well as in the glycolytic pathway (Nguyen et al., 2016).

Here we investigated a kinase with similarity to YPK1 type kinases, which turned out to be unique in *Trichoderma* spp. With functions in regulation of genes within the SOR cluster, but also in cellulase gene expression, this kinase may represent an interesting evolutionary adaptation of *T. reesei* to the need of balanced resource distribution between nutrient acquisition and reaction to competitors.

## Results

### A Unique AGC Family Protein Kinase in the Vicinity of the SOR Cluster

Searching for signaling factors modulating expression of the SOR cluster, we found an AGC family serine/threonine protein kinase represented by the gene model TR_53776 in its vicinity (scaffold 1:478516-479682; 2.3 kb upstream of TR_73618/*pks11*), which may regulate transcription factor activity or influence enzyme stability by phosphorylation. A recent study on protein phosphorylation in *T. reesei* (Nguyen et al., 2016) revealed phosphorylation of the transporter within the SOR cluster (TR_43701), which impacts secondary metabolite levels (Monroy et al., 2017). TR_53776 is related to YPK1 type kinases (protein domain cd11651), which are essential for cell growth in yeast and play a role in endocytosis and cell wall integrity. The *ypk1* homolog in *Neurospora crassa* (NCU07280) was found to be essential (Park et al., 2011), but analyses of conditional mutants in *Aspergillus nidulans* showed functions in growth, hyphal morphogenesis, and delivery of cell membrane and cell wall constituents to the hyphal apex (Colabardini et al., 2013). TR_53776 comprises one potential PEST motif (amino acids 6–24) in its sequence, which indicates regulation of protein stability by phosphorylation (Rechsteiner and Rogers, 1996) as well as eight poor PEST motifs. TargetP 2.0, NetNES 1.1, and WoLF PSORT were used for prediction of localization; however, no targeting sequences to the nucleus or other cellular compartments were detected. Evaluation of available transcriptome data revealed that TR_53776 is regulated in an induction specific manner (Stappler et al., 2017a), but no significant regulation was observed by light, photoreceptors or CRE1 (Tisch et al., 2011b; Tisch and Schmoll, 2013; Monroy et al., 2017).

**FIGURE 1:**
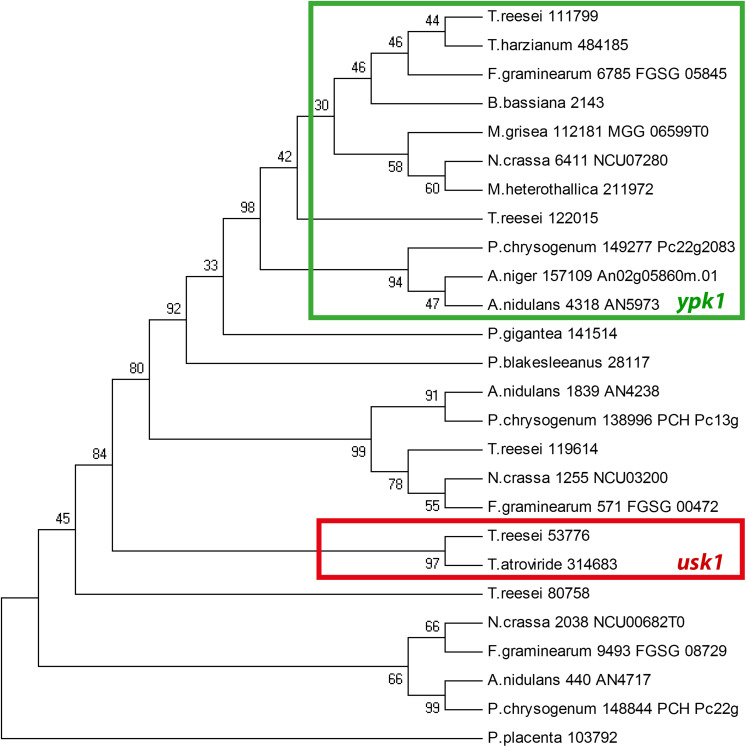
Phylogenetic analysis of *usk1* in fungi. The phylogenetic tree was obtained using MEGA4.0 with the maximum parsinomy method. Numbers at branches indicate bootstrap support values. Protein IDs or locus IDs are provided along with species names for sequences originating from *Trichoderma reesei*, *Trichoderma harzianum*, *Fusarium graminearum*, *Beauveria bassiana*, *Magnaporthe grisea*, *Neurospora crassa*, *Myceliophthora heterothallica*, *Penicillium chrysogenum*, *Aspergillus niger*, *Aspergillus nidulans*, *Phlebiopsis gigantea*, *Phycomyces blakesleeanus*, and *Postia placenta*.

We further found that the genomic localization of TR_53776 close to the SOR cluster is not syntenic in other fungi such as *Aspergillus niger* or *P. chrysogenum*. Accordingly, bidirectional best hit analysis showed that the best blast hits of TR_53776 in these fungi were related to kinases other than TR_53776. Therefore, we performed a phylogenetic analysis of TR_53776 and its most closely related kinases in *T. reesei* using the best hits of related fungi along with putative homologs from more distantly related fungi. This analysis showed that TR_53776 is not conserved in fungi, not even in closely related *Trichoderma* spp. like *Trichoderma harzianum* (Figure 1). Only for *Trichoderma atroviride*, a homolog was detected. Consequently, we named TR_53776 *u*nique *S*OR cluster *k*inase 1 (*usk1*).

### Evaluation of a Relevance of *usk1* for Growth

While *usk1* is not an ortholog of the well characterized *ypk1* kinases, its close relationship still made us evaluate whether there are functional similarities by deleting *usk1* in QM6a. In contrast to YPK1 homologs in *N. crassa* and *A. nidulans*, *T. reesei* USK1 is not essential for growth. Strains lacking *usk1* are viable and analyses of hyphal extension on solid media with malt extract, cellulose, or glucose did not show any growth defect (Supplementary Figures S1, S2). We also tested the relevance of USK1 for dealing with osmotic stress including ion stress (minimal media with 1 M NaCl or 1 M sorbitol) upon growth in the presence of cellulose or glucose in light and darkness. However, also under these conditions, no significant alterations in growth compared to wild-type was observed (Supplementary Figures S1, S2).

### USK1 Positively Impacts Cellulase Gene Expression

**FIGURE 2:**
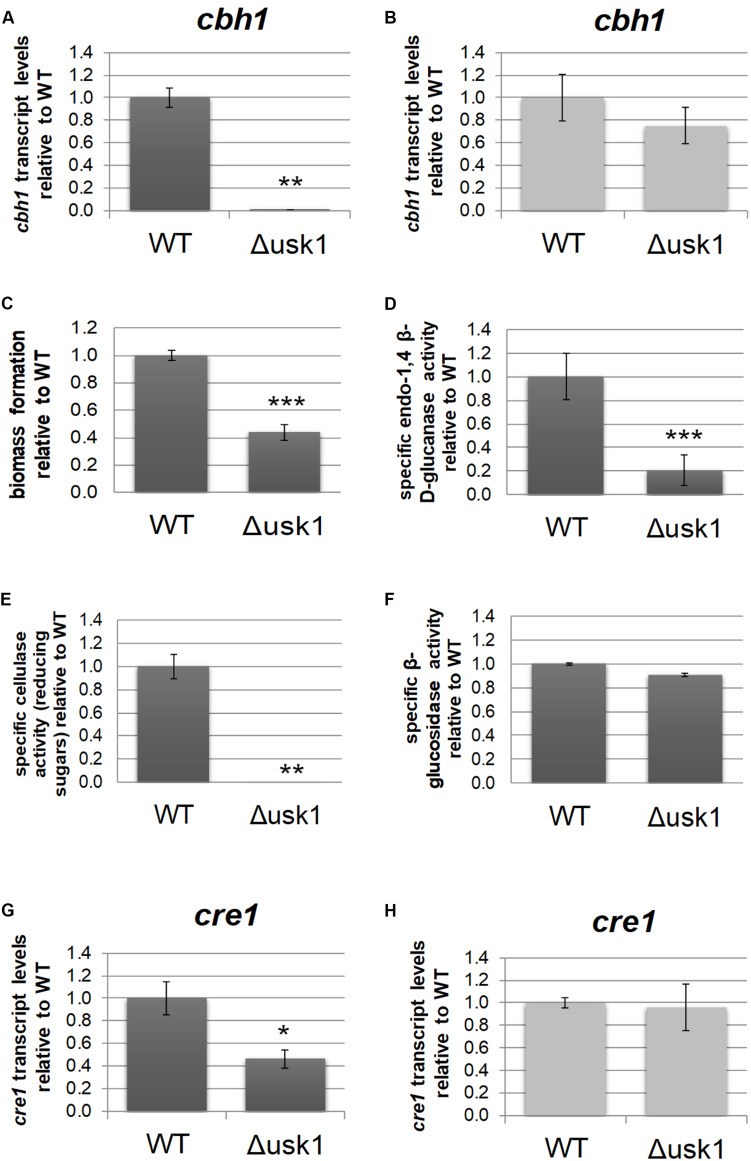
Regulatory impact of USK1 on cellulase regulation and on CRE1. Transcript levels of cellobiohydrolase 1 (*cbh1*) as representative cellulase are shown for growth on microcrystalline cellulose in constant darkness (**A**; DD) and constant light (**B**; LL). **(C)** Biomass formation upon growth on microcrystalline cellulose in constant darkness. **(D)** Specific endo-1,4 β-D-glucanase activity upon growth on cellulose in darkness. **(E)** Specific cellulase activity (reducing sugars) upon growth on cellulose in darkness. **(F)** Specific β-glucosidase activity upon growth on cellulose in darkness. **(G,H)** Transcript abundance of *cre1* upon growth on cellulose in constant darkness **(G)** or constant light **(H)**. Error bars indicate standard deviations of three biological replicates. Asterisks indicate statistically significant differences compared to wildtype (*p-value < 0.05, **p-value < 0.01, ***p-value < 0.001).

Transcript levels of *usk1* are upregulated in an induction specific manner (Stappler et al., 2017a). Therefore, we evaluated a relevance of this kinase for regulation of cellulase gene expression. Analysis of transcript levels of *cbh1* upon growth on minimal medium with cellulose as carbon source revealed a positive impact of USK1 on *cbh1* transcript abundance in darkness (Figure 2A), but not in light (Figure 2B). Accordingly, biomass formation of Δ*usk1* in liquid media with cellulose as carbon source decreased considerably in darkness (Figure 2C) and specific secreted endo-β-1,4-glucanase activity dropped (Figure 2D). Accordingly, also liberation of reducing sugars from cellulose, representing cellulase activity, decreased (Figure 2E). However, β-glucosidase activity remained largely unchanged in.Δ*usk1* (Figure 2F). In light only a minor alteration in biomass formation (83 ± 5.4%) was observed between mutant and wildtype. Specific endo-β-1,4-glucanase activity representing cellulase activity remained below detection limits in the deletion strain, as in the wildtype upon growth in constant light.

Since CRE1 was previously shown to impact the SOR cluster (Monroy et al., 2017) besides its function as a carbon catabolite repressor (Strauss et al., 1995), we tested an influence of USK1 on transcript abundance of *cre1*. In darkness, we observed a positive effect of USK1 (about twofold) on transcript levels of *cre1* on cellulose (Figure 2G), while no effect was seen in light (Figure 2H).

### USK1 Is Involved in Regulation of Secondary Metabolism

Due to its genomic position close to the SOR cluster, we analyzed a potential influence of USK1 on gene regulation of secreted metabolites in general, of those in the SOR cluster and the impact on production of known secondary metabolites produced by *T. reesei*.

**FIGURE 3:**
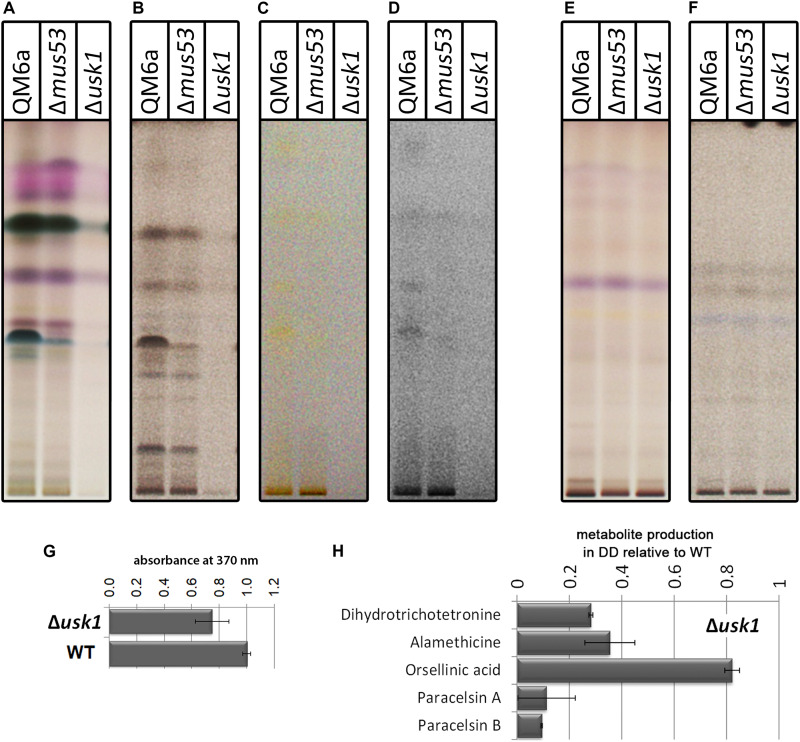
Impact of USK1 on production of secondary metabolites and their regulators. Wildtype QM6a, parental strain QM6aΔ*mus53* and Δ*usk1* were grown on minimal medium in constant light (LL; **E,F**) or constant darkness (DD; **A–D**) with cellulose as carbon source. The individual panels show different visualization techniques showing different sets of secondary metabolites of substance classes responsive to the respective technique. **(A,E)** show visualization at visible light after derivatization with anisaldehyde solution. **(B,F)** show absorption at 254 nm after development. **(C,D)** show visualization at visible light after development. For better visibility of the yellow compounds shown in **(C)** in the saturation was lowered in **(D)**. Analyses were done in biological triplicates and are related to biomass values. **(G)** Determination of absorbance at 370 nm in the supernatant in relation to the wildtype in constant darkness. Analysis was done in biological triplicates. **(H)** Quantitative analyses by mass spectrometry with internal standards were performed from strains grown on cellulose in darkness. Abundance of the mentioned metabolites is shown in relation to the wildtype. Values are normalized to biomass.

First we tested for a general regulation of secreted metabolite patterns by analysis of secreted compounds during cultivation on minimal medium with cellulose as carbon source by high performance thin layer chromatography (HPTLC) (Hinterdobler et al., 2019). This method provides an informative visual overview on abundance of metabolites in multiple samples and represents a valuable alternative to mass spectrometry, particularly for organisms with only few compounds already known and characterized like *T. reesei* (Frisvad et al., 2018; Hinterdobler et al., 2019). We found a clear decrease in overall abundance of metabolites upon growth in liquid culture with cellulose as carbon source in darkness in Δ*usk1* (Figures 3A–D), but no changes in light compared to wildtype under the same conditions (Figures 3E,F). Although biosynthesis of yellow pigments, largely representing sorbicillinoids, is at a low level under these conditions, the positive effect of USK1 on production of individual compounds of this group is still visible (Figures 3C,D). Accordingly, analysis of the supernatant for absorbance at 370 nm, representing part of the sorbicillinoids, is decreased (Figure 3G).

Quantification of selected secondary metabolites in the supernatant of cellulose grown cultures by mass spectrometry with internal standards revealed decreased levels for the sorbicillin derivative dihydrotrichotetronine (syn. bislongiquinolide or bisorbibutenolide, Shirota et al., 1997; a major product of the SOR cluster, Monroy et al., 2017), alamethicine, orsellinic acid, and paracelsin (Figure 3H). Consequently, USK1 does not only impact sorbicilline compounds associated with the SOR cluster, such as dihydrotrichotetronine, but also other compounds, which is in agreement with HPTLC analysis. Hence, this putative kinase is not specifically targeting sorbicillin production, but presumably impacts regulation of multiple secondary metabolite gene clusters to modulate secondary metabolism more broadly.

**FIGURE 4:**
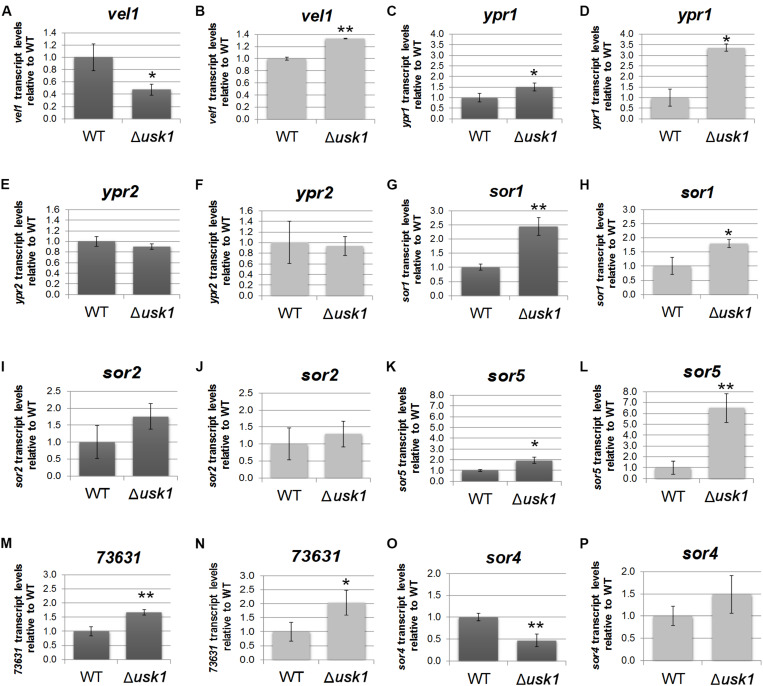
Influence of USK1 on regulators of secondary metabolism and the SOR cluster. Evaluation of the impact of USK1 on known regulators of secondary metabolism. Transcript abundance of *vel1* **(A,B)**, *ypr1* **(C,D)**, and *ypr2* **(E,F)** upon growth on cellulose in constant darkness (DD; **A,C,E**) or constant light (LL; **B,D,F**). **(G–P)** Impact of USK1 on SOR cluster genes. Transcript levels of sor1 (TR_73618; **G,H**), sor2 (TR_73621; **I,J**), sor5 (TR_73623; **K,L**), TR_73631 **(M,N)**, and sor4 (TR_43701; **O,P**) are shown for growth on microcrystalline cellulose in constant darkness (DD; **G,I,K,M,O**) and constant light (LL; **H,J,L,N,P**). Error bars indicate standard deviations of three biological replicates. Asterisks indicate statistically significant differences compared to wildtype (*p-value < 0.05, **p-value < 0.01).

In order to gain insight into the regulatory basis of the effect of USK1 on secondary metabolite production, we analyzed its impact on transcript abundance of the general regulator of secondary metabolism, *vel1*, and the two transcription factor genes associated with the SOR cluster, *ypr1* and *ypr2*. We found that USK1 positively regulates transcript abundance of *vel1* in darkness, but slightly negatively in light (Figures 4A,B). For *ypr1*, we found negative regulation by USK1 in light and darkness (Figures 4C,D), but no effect on *ypr2* (Figures 4E,F).

Screening for an influence of USK1 on transcript abundance of SOR cluster genes (Figures 4G–P) showed a negative effect USK1 on *sor1* (TR_73618), *sor5* (TR_73623), and TR_73631 in light and darkness. The gene encoding the transporter SOR4 (TR_43701) is positively regulated in darkness by USK1 (Figure 4O). While USK1 appears to exert a negative effect on transcript levels of several SOR cluster genes, the decreased abundance of secondary metabolites associated with this cluster in the culture filtrate as shown in Figures 3C,D,G may be due to the decreased levels of the transporter TR_43701 (Figure 4O).

## Discussion

As one of the most important biotechnological workhorses among filamentous fungi, the physiology of *T. reesei* and particularly its secondary metabolism are of high interest to industry. It is of utmost importance to ensure the lack of harmful secondary metabolites in food and feed products from fungal fermentations. Therefore, investigation of regulators and the conditions under which they are operative is crucial to understand secondary metabolism in *T. reesei*.

Protein kinases generally have broad functions in fungi, including regulation of pathogenicity and virulence, stress response, circadian rhythmicity, development, and metabolism (Dickman and Yarden, 1999; Diernfellner and Schafmeier, 2011; Brown et al., 2014; Turra et al., 2014; Manfiolli et al., 2019). The kinomes of fungi share similarity, albeit domain distribution and density of kinomes are suggested to reflect taxonomy (Kosti et al., 2010). In *A. nidulans*, protein kinases were shown to impact secondary metabolism, particularly the group of mitogen activated protein kinases (De Souza et al., 2013). MAPkinases impact secondary metabolism also in *Podospora anserina* (Bidard et al., 2012) and *N. crassa* (Park et al., 2008). USK1 is related to YPK-type protein kinases (particularly to YPK2) which are essential for cell growth and maintenance of cell wall integrity (Chen et al., 1993; Roelants et al., 2002). Saccharomyces *cerevisiae* YPK2 is subject to phosphorylation by TOR kinases, thereby impacting actin polarization (Kamada et al., 2005). Moreover, YPK2 is implicated in the sphingolipid mediated signaling pathway which targets multiple physiological functions like endocytosis, ubiquitin dependent proteolysis, regulation of nutrient uptake, the cell cycle, translation, and heat stress response (Chung et al., 2001; Cowart and Obeid, 2007). The *N. crassa* kinase most closely related to USK1 is NCU07280/STK50, which is an essential gene in this fungus (Park et al., 2011) and only shows minor regulation in response to phytosphingosine (Videira et al., 2009). A function in glucose starvation was reported for the fission yeast kinase related to USK1, GAD8 (Saitoh et al., 2015). Also in *A. nidulans*, the most closely related kinase AN5973 is an essential gene required for polar axis establishment and germling growth (Colabardini et al., 2013; De Souza et al., 2013).

Although USK1 is closely related to these kinases, our analyses revealed that it represents a unique kinase in *Trichoderma* spp. Accordingly, deletion of *usk1* also was not fatal and growth characteristics of Δ*usk1* were largely normal. Nevertheless, USK1 is highly relevant for expression of cellulolytic enzymes and impacts regulation of genes of the SOR cluster, which is in its genomic vicinity. In Trichoderma *parareesei*, with the closest homology to *T. reesei* this localization is conserved, but already in *Trichoderma citrinoviride*, the locus of *usk1* and the SOR cluster is altered as it is in *T. atroviride* and *Trichoderma gamsii*. This is in agreement with the SOR cluster being only present in the section Longibrachiatum (Druzhinina et al., 2016). Consequently, the regulatory impact of USK1 on the SOR cluster could have been a beneficial event after acquisition by lateral gene transfer.

Cellulase gene expression is regulated in response to diverse environmental cues, the most important of them being the kind of carbon source and its abundance as well as the impact of light. This regulation is influenced broadly by components of different signal transduction pathways, which are known to be subject to modifications in activity levels by phosphorylation (Bazafkan et al., 2014; Paloheimo et al., 2016; Schmoll, 2018). Transcript levels of *usk1* are decreased upon growth in darkness on glucose compared to cellulose in QM6a (Beier, Hinterdobler, and Schmoll, unpublished). Additionally, *usk1* showed induction specific regulation (Stappler et al., 2017a). Considering the substantial impact of USK1 on biomass formation and cellulase production upon growth on cellulose, it can be assumed that the major function of USK1 is in nutrient signal transduction and potentially in its coordination with regulation of secondary metabolism. Thereby, the impact of USK1 is not limited to the genes of the cluster and the compounds associated with the SOR cluster. With a relevance of USK1 also to the production of alamethicine and paracelsine, a broader function of USK1 on secondary metabolism is obvious, which is also reflected in considerably decreased levels of secreted metabolites detected in the HPTLC analysis (Figure 3). These findings are in agreement with previous reports, showing that alterations in the levels SOR cluster compounds are often accompanied with modulations of further metabolites associated with other secondary metabolite clusters (Bazafkan et al., 2017; Monroy et al., 2017; Dattenböck et al., 2018). Also for the transcription factor YPR2, which regulates the SOR cluster (Derntl et al., 2016; Monroy et al., 2017), regulation of multiple genes involved in secondary metabolism was shown (Hitzenhammer et al., 2019). For the transcription factor XPP1, which exerts functions in primary and secondary metabolism, a broad impact on both quantity and diversity of secreted metabolites was shown as well (Derntl et al., 2017b). While the actual target(s) of USK1 remain to be elucidated, it can be assumed that in agreement with the results for YPR2 and XPP1, regulation of secondary metabolism is governed by a flat hierarchical network that targets gene regulation broadly rather than specifically, hence establishing a complex network of interdependent regulatory effects. The modulating function of USK1 on the SOR cluster genes and their products as well as on enzyme production moreover add further support for a crosstalk between enzyme expression and secondary metabolism in *T. reesei*.

## Materials and Methods

### Strains and Cultivation Conditions

QM6a (ATCC13631) (Martinez et al., 2008) and QM6aΔ*mus53* (Steiger et al., 2011) was used in this study. For analysis of gene regulation by USK1, strains were cultivated in constant darkness (to exclude interference by circadian rhythmicity) on malt extract agar plates for 14 days to produce conidia for inoculation.

For liquid cultures 10^8^ conidia/L were used for inoculation. Strains grown in Mandels Andreotti minimal medium (Mandels and Andreotti, 1978) with 1% (w/v) cellulose (avicel, SERVA, Alfa Aesar, Karlsruhe, Germany) in constant light (1700 lux, Osram L18W/835 fluorescent bulbs) or in constant darkness at 28°C at 200 r/min. Mycelia and supernatants were harvested after 96 h. Red safety light (darkroom lamp, Philips PF712E, red, E27, 15 W) was applied to avoid random light pulses during harvesting of dark grown cultures.

Hyphal extension was investigated on plates in constant light or constant darkness on the carbon sources mentioned with the experiment and three biological replicates were used. Sexual development was analyzed with strains grown on malt extract media at 22°C in dark-light cycles as described previously (Schmoll, 2013).

### Construction of Δ*usk1*

Deletion of *usk1* was done in QM6aΔ*mus53* (deficient in non-homologous end joining) using the streamlined procedure described previously (Schuster et al., 2012) with the *hph* marker cassette. Transformation was done by protoplasting (Gruber et al., 1990). Successful deletion was confirmed by PCR using primers 53776RTF and 53776RTR. Sequences of all primers used in this study are given in Supplementary Table S1. Copy number determination for integration of the deletion cassette in to the genome of *T. reesei* was performed as described previously (Tisch and Schmoll, 2013). This analysis showed integration of one single copy of the *usk1* deletion cassette into the genome and hence confirmed homologous integration (Supplementary Figure S3).

### Nucleic Acid Manipulation and Analysis

Isolation of total RNA, quality control, and RTqPCR were done as described previously (Tisch et al., 2011a; Bazafkan et al., 2017). *Sar1* was used as reference gene for RT-qPCR as it was shown to be generally very stable across different conditions (Bazafkan et al., 2017). We considered three biological and three technical replicates for analysis using the CFX Maestro analysis software (Biorad, Hercules, CA, United States). Primers used for analysis are listed in Supplementary Table S1.

### Enzyme Assays

Endo-1,4-β-glucanase activity in culture filtrates was determined using azo-CM-cellulose (S-ACMC-L, Megazyme, Wicklow, Ireland) according to the manufacturers instructions.

Cellulase activity as represented by reducing sugars was measured using the pHBAH (*p-*hydroxybenzoic acid hydrazide) assay as described previously (Mellitzer et al., 2012). Therefore, 1% (w/v) cellulose (Alfa Aesar, Karlsruhe, Germany) served as sole carbon source in the substrate suspension. The absorbance at 410 nm was determined in 96-well flat bottom plates (Greiner Bio One, Kremsmünster, Austria) using a BioTek Synergy^TM^ Mx microplate reader.

Analysis of β-glucosidase was performed using the chromogenic substrate 4-nitrophenyl-β-D-glucopyranosid as described previously (da Silva Delabona et al., 2017).

Biomass for determination of specific enzyme activity was measured in the presence of insoluble cellulose with intracellular protein content as representative criterium as described previously (Schuster et al., 2011; Stappler et al., 2017b). Three biological replicates and technical triplicates were analyzed.

### Bioinformatic Analysis

Phylogenetic analysis was performed as described previously (Bazafkan et al., 2015) using the software packages ClustalX1.82 and MEGA4 (Tamura et al., 2007). Therefore, sequences were obtained from JGI Mycocosm. Homology and BLAST searches were performed on the JGI Mycocosm platform^1^. Screening for PEST domains (Rechsteiner and Rogers, 1996) was done using the online resource ePESTFIND^2^ with default settings. TargetP 2.0 (Emanuelsson et al., 2007), NetNES 1.1 (la Cour et al., 2004), and WoLF PSORT (Horton et al., 2007) were applied to analyze protein characteristics. For detection of conserved domains, the conserved domain search at NCBI^3^ (Marchler-Bauer et al., 2017). Statistical significance of values was evaluated by *t*-test in RStudio (compare_means, ggpubr).

### Analysis of Secondary Metabolites Secreted by *T. reesei*

High performance thin layer chromatography was performed as described previously from culture supernatants of cellulose grown mycelia by extraction with chloroform (Monroy et al., 2017; Hinterdobler et al., 2019). Measurements are related to biomass abundance for every sample.

Abundance of selected secondary metabolites was analyzed by mass spectrometry with internal standards as described previously (Malachova et al., 2014; Monroy et al., 2017).

## Data Availability Statement

All datasets relevant for this study are included in the article/Supplementary Material.

## Author Contributions

SB contributed to analysis of growth and cellulase production. WH contributed to analysis of secondary metabolite production. AM participated in secondary metabolite analysis and cultivation. HB constructed the recombinant strain. SB and WH participated in drafting the manuscript. MS conceived of the study, participated in analysis and interpretation of results, and wrote the final version of the manuscript. All authors read and agreed to publication of the final manuscript.

## Conflict of Interest

The authors declare that the research was conducted in the absence of any commercial or financial relationships that could be construed as a potential conflict of interest.

## Footnotes

1 https://mycocosm.jgi.doe.gov/mycocosm/home

2 http://emboss.bioinformatics.nl/cgi-bin/emboss/epestfind

3 https://www.ncbi.nlm.nih.gov/Structure/cdd/wrpsb.cgi?
