# Supplementary material for: The Kinase USK1 Regulates Cellulase Gene Expression and Secondary Metabolite Biosynthesis in Trichoderma reesei
Source: Front Microbiol. 2020 May 20;11:974. doi: 10.3389/fmicb.2020.00974 (PMC7251307; doi:10.3389/fmicb.2020.00974)
Supplement: Supplementary file 1 [file Data_Sheet_1.PDF]

The kinase USK1 regulates cellulase gene expression and secondary metabolites in *Trichoderma reesei*

Sabrina Beier[1], Wolfgang Hinterdobler[1], Alberto Alonso Monroy[1], and Schmoll Monika[1]*

Supplementary material

Figure S1.

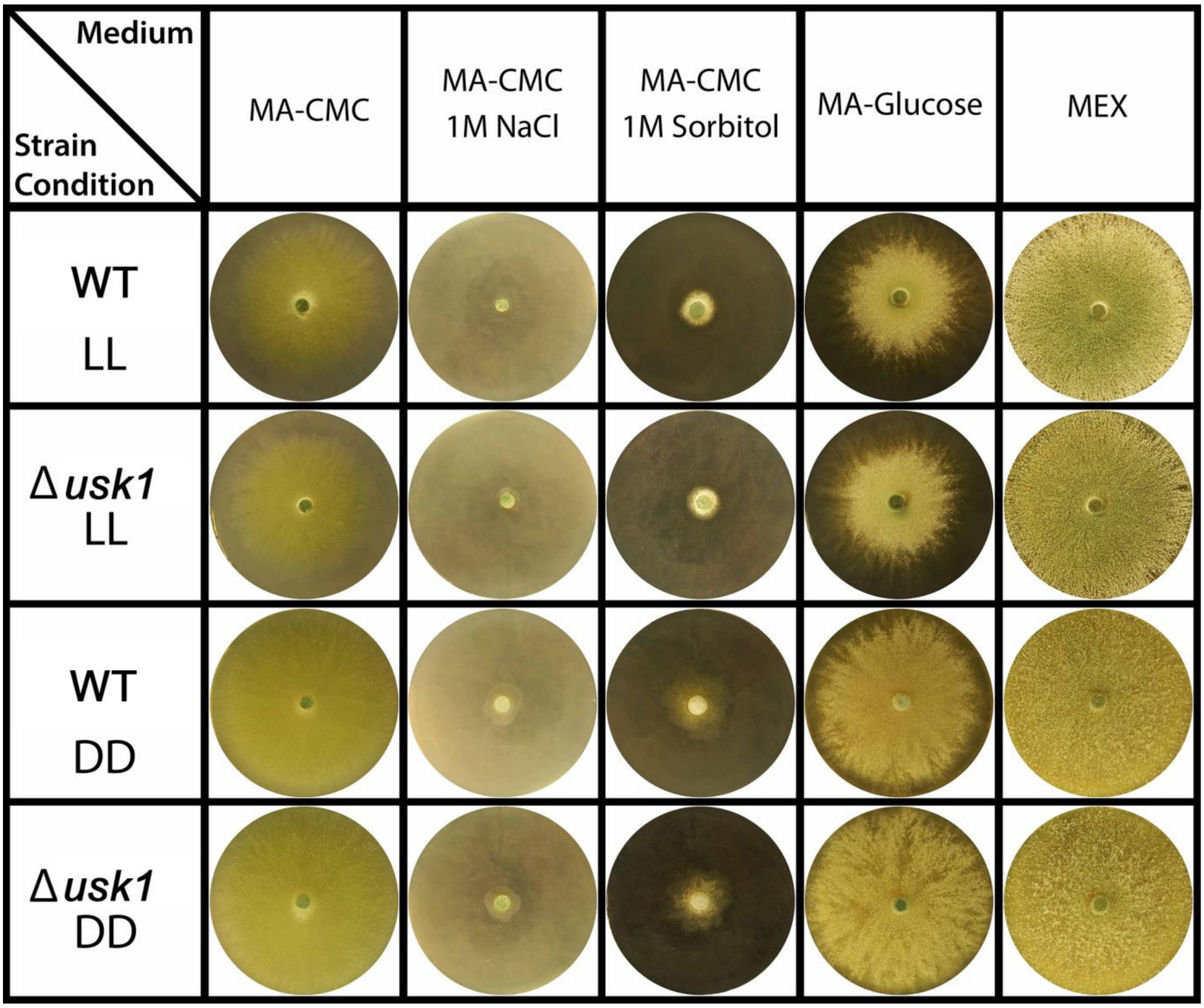

Supplementary figure 1. Growth characteristics of wild-type and recombinant strains. Strains were grown Mandels Andreotti minimal medium (MA) on 1 % (w/v) carboxymethylcellulose (CMC), 1 % (w/v) glucose or on 3 % (w/v) malt extract (MEX) in constant light (LL) or constant darkness (DD). In order to evaluate behavior under osmotic stress conditions, 1 M NaCl or 1 M sorbitol were added to plates containing CMC, which were otherwise treated equally.

Figure S2.

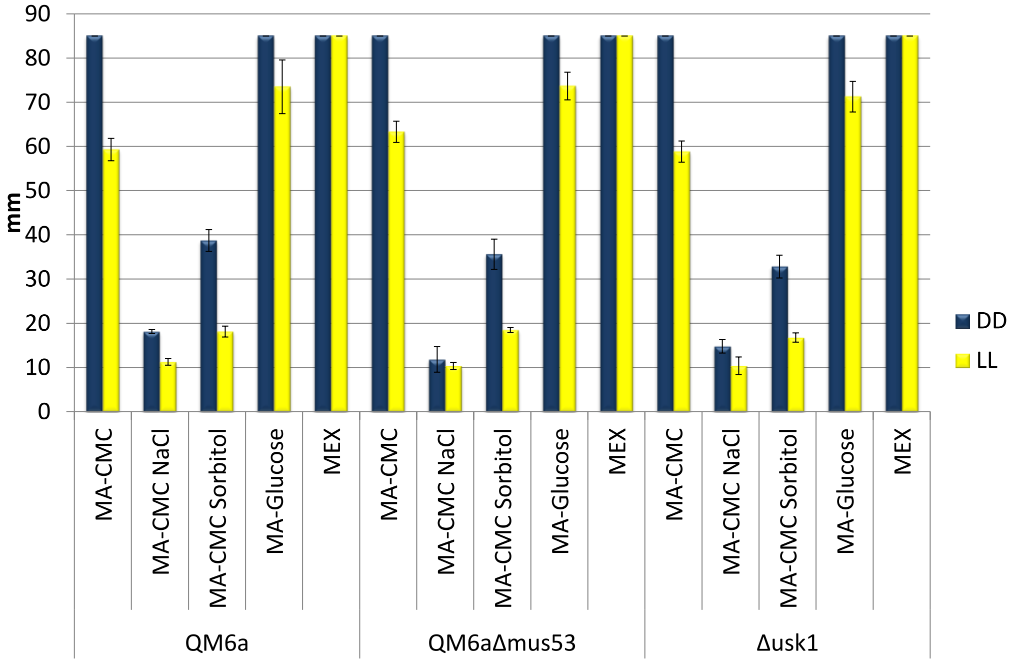

Supplementary figure 2. Evaluation of hyphal extension of wild-type and recombinant strains. Strains were grown Mandels Andreotti minimal medium (MA) on 1 % (w/v) carboxymethylcellulose (CMC), 1 % (w/v) glucose or on 3 % (w/v) malt extract (MEX) in constant light (LL) or constant darkness (DD). In order to evaluate behavior under osmotic stress conditions, 1 M NaCl or 1 M sorbitol were added to plates containing CMC, which were otherwise treated equally.

Figure S3

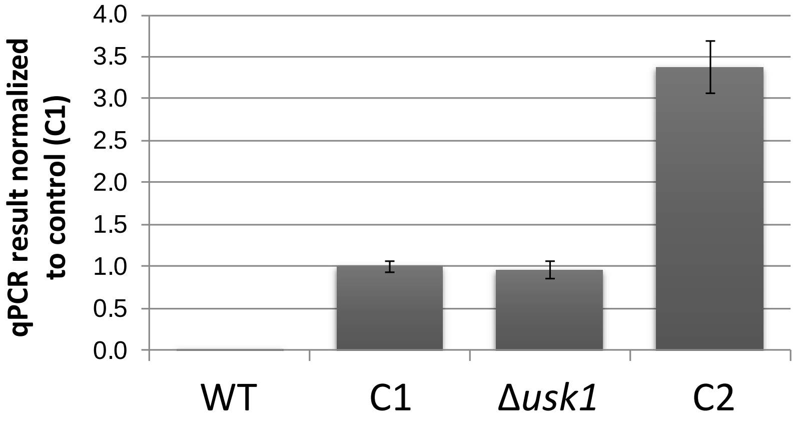

Supplementary figure S3. Determination of copy number of deletion cassette(s) in strain Δ*usk1*. Copy number was determined by quantitative PCR according to Tisch et al., 2011, with the controls described there. Amplicon levels of *l6e* were used as internal controls for DNA amounts. Strains with confirmed copy number were taken as controls. Control strain C1 contains one copy of the *hph* deletion casette and control strain C2 contains three copies of the *hph* deletion cassette. The parental strain of Δ*usk1* was used as negative control and showed no signal. The amplicon of Δ*usk1* showed the same abundance as that of control C1 and hence the copy number of one was confirmed.

Supplementary Table S1. Oligonucleotides used in this study.

name	purpose	sequence	Protein ID	target gene	Reference
53776_3F	deletion cassette	5' CTCCTTCAATATCATCTTCTGTCTCCG ACCTATAGTGCTAGACCCCAGC 3'	53776	*usk1*	Schuster et al., (2012)
53776_3R	deletion cassette	5' GCGGATAACAATTTCACACAGGAAA CAGCATCTAGCCGTGATACTCTGG 3'	53776	*usk1*	Schuster et al., (2012)
53776_5F	deletion cassette	5' GTAACGCCAGGGTTTTCCCAGTCACG ACGACACTCACCTTCTCATCTCC 3'	53776	*usk1*	Schuster et al., (2012)
53776_5R	deletion cassette	5' ATCCACTTAACGTTACTGAAATCTC CAACGACTCCTCCACTTACATTCC 3'	53776	*usk1*	Schuster et al., (2012)
53776RTF	RT-qPCR/ transformant screening	5' GCGCACTGGAGTATCTGCACGA 3'	53776	*usk1*	Monroy et al., (2017)
53776RTR	RT-qPCR/ transformant screening	5' CGGTAGCCCTGTCAGCATCTCG 3'	53776	*usk1*	Monroy et al., (2017)
RTcbh1F	RT-qPCR	5' ACCGTTGTCACCCAGTTCG 3'	123989	*cbh1*	Tisch et al., (2011)
RTcbh1R	RT-qPCR	5' ATCGTTGAGCTCGTTGCCAG 3'	123989	*cbh1*	Tisch et al., (2011)
RT_VEL_R1	RT-qPCR	5' GCAGGAACACCAGTCAGGATG 3'	122284	*vel1*	Bazafkan et al., (2015)
RT_VEL_F1	RT-qPCR	5' CGAGGAGGGCAAGGACATTAC 3'	122284	*vel1*	Bazafkan et al., (2015)
RT_102499_F	RT-qPCR	5' CTTGATGGCGATGGCTTGTA 3'	102499	*ypr1*	this study
RT_102499_R	RT-qPCR	5' GCGATGGCATTAGGGAAGAA 3'	102499	*ypr1*	this study
int_102497_F1	RT-qPCR	5' TGCCTTGGTGGTACCGGGTCTA 3'	102497	*ypr2*	Monroy et al., (2017)
int_102497_R1	RT-qPCR	5' TCGTCCAAAGCCATGATGCCGT 3'	102497	*ypr2*	Monroy et al., (2017)
RT_73618_F	RT-qPCR	5' ATGACGAGGATAGCAAGGCGGC 3'	73618	*sor1*	Monroy et al., (2017)
RT_73618_R	RT-qPCR	5' AATGGACAACTCTGCTCCCGCC 3'	73618	*sor1*	Monroy et al., (2017)
RT_73621_F	RT-qPCR	5' GCAACCTCGTCGATTTGGCTGC 3'	73621	*sor2*	Monroy et al., (2017)
RT_73621_R	RT-qPCR	5' AAGTGTCTCGAGAAGGACGCGC 3'	73621	*sor2*	Monroy et al., (2017)
RT_73623_F	RT-qPCR	5' GACGAGGATGACGTGAAGCGCT 3'	73623	*sor5*	Monroy et al., (2017)
RT_73623_R	RT-qPCR	5' GCCAAGACCAGCGAGTCTTCCA 3'	73623	*sor5*	Monroy et al., (2017)
RT_73631_F	RT-qPCR	5' AACGGCTCCGAAATCACTGCGA 3'	73631		Monroy et al., (2017)
RT_73631_R	RT-qPCR	5' CCCCGGCATCAGATATCGCAGG 3'	73631		Monroy et al., (2017)
RT_43701_F	RT-qPCR	5' GTCAGCACCATTTGGCTTCGGC 3'	43701	*sor4*	Monroy et al., (2017)
RT_43701_R	RT-qPCR	5' TCCCTGACAACTGCGCCATAGC 3'	43701	*sor4*	Monroy et al., (2017)
SAR RTF1	RT-qPCR	5' TGGATCGTCAACTGGTTCTACGA 3'	61470	*sar1*	Steiger et al., (2010)
SAR RTR1	RT-qPCR	5' GCATGTGTAGCAACGTGGTCTTT 3'	61470	*sar1*	Steiger et al., (2010)

References

Bazafkan, H., et al., 2015. Mating type dependent partner sensing as mediated by VEL1 in *Trichoderma reesei*. Mol Microbiol. 96, 1103-18.

Monroy, A. A., et al., 2017. A CRE1- regulated cluster is responsible for light dependent production of dihydrotrichotetronin in *Trichoderma reesei*. PLoS One. e0182530.

Schuster, A., et al., 2012. A versatile toolkit for high throughput functional genomics with *Trichoderma reesei*. Biotechnol Biofuels. 5, 1.

Steiger, M. G., et al., 2010. An accurate normalization strategy for RT-qPCR in *Hypocrea jecorina* (*Trichoderma reesei*). J Biotechnol. 145, 30-7.

Tisch, D., et al., 2011. The phosducin-like protein PhLP1 impacts regulation of glycoside hydrolases and light response in *Trichoderma reesei*. BMC Genomics. 12, 613.
